# An interim analysis of the influence of eating disorder risk, body dissatisfaction, physical activity, and diet on bone mineral density in college-aged females

**DOI:** 10.1080/15502783.2025.2550169

**Published:** 2025-11-04

**Authors:** Leah E. Allen, Christy V. Le, Conor F. Horlock, Margaret T. Jones, Andrew R. Jagim, Jennifer B. Fields

**Affiliations:** a University of Connecticut, Department of Nutritional Sciences, Storrs, CT, USA; b George Mason University, Frank Pettrone Center for Sports Performance, Fairfax, VA, USA; c George Mason University, Sport, Recreation, and Tourism Management, Fairfax, VA, USA; d Mayo Clinic Health System, Sports Medicine, Onalaska, WI, USA

**Keywords:** Bone health, physical activity, college female, body image

## Abstract

**Background:**

This study aimed to examine relationships between BMD z-scores, eating disorder (ED) risk, body image dissatisfaction (BID), physical activity, and dietary intake in college females.

**Methods:**

College-aged females (*n* = 20, age: 19 ± 1 yrs, weight: 58 ± 9 kg, BF%: 29 ± 6%) participated in this cross-sectional study. Whole body, total hip, femoral neck, and lumbar spine BMD were assessed using dual-energy x-ray absorptiometry. Participants completed validated questionnaires to assess ED risk and BID. Objective physical activity (daily steps, daily physical activity energy expenditure (PAEE), time spent in sedentary (PA_s_), light (PA_L_), moderate (PA_M_), and vigorous (PA_V_) zones), and dietary intake were quantified across 3-days via a wrist-worn accelerometer and a self-reported food log, respectively. Spearman correlations evaluated relationships between BMD, ED scores, BID, physical activity metrics, and dietary intake (*p* < 0.05).

**Results:**

47–65% of participants had low BMD (z-score < 0) at whole-body, hip, and spine sites, with 40% classified as osteopenic (Ta ble 1). Additionally, 20% were at risk of ED and 65% conveyed BID. Participants averaged 15,789 ± 4,116 daily steps, expending 986 ± 396 active calories. Total hip BMD z-scores were positively associated with daily steps (*r* = 0.462, *p* = 0.046), PAEE (*r* = 0.493, *p* = 0.032), and PA_M_ (*r* = 0.575, *p* = 0.010). No other relationships were observed between BMD, ED risk, BID, and dietary intake (calories, carbohydrates, protein, fat, calcium) (*p* > 0.05).

**Conclusions:**

A high incidence of low BMD was observed across all sites (whole body, total hip, femoral neck, and lumbar), with BMD z-scores associated with physical activity (daily step counts, PAEE, and PA_M_), but not with dietary intake, ED risk, or BID. These findings highlight the importance of physical activity for maintaining bone health in premenopausal females. As BMD reflects long-term bone health, it may not capture acute dietary or lifestyle changes, highlighting the need to assess dynamic bone turnover markers.

**Table 1.** Body composition, bone mineral density (BMD) z-scores, and prevalence of negative BMD z-scores.

	BMD z-Score (95% CI)	% of Participants with Low BMD (z-score < 0)	% Osteopenia
Whole Body	0.1 ± 1.0 (−0.4,0.6)	65% (*n* = 13)	10% (*n* = 2)
Total Hip	0.1 ± 1.0 (−0.4,0.7)	47% (*n* = 9)	16% (*n* = 3)
Femoral Neck	0.1 ± 1.2 (−0.5,0.6)	53% (*n* = 10)	21% (*n* = 4)
Total Lumbar Spine	−0.1 ± 1.1 (−0.6,0.4)	65% (*n* = 13)	30% (*n* = 6)

Values are mean±SD (95% CI).

